# Genetic Blockade of NAAA Cell-specifically Regulates Fatty Acid Ethanolamides (FAEs) Metabolism and Inflammatory Responses

**DOI:** 10.3389/fphar.2021.817603

**Published:** 2022-01-07

**Authors:** Xiaohua Xie, Yitian Li, Sennan Xu, Pan Zhou, Longhe Yang, Yaping Xu, Yan Qiu, Yungang Yang, Yuhang Li

**Affiliations:** ^1^ Department of Pediatrics, The First Affiliated Hospital of Xiamen University, Xiamen, China; ^2^ School of Medicine, Institute of Pediatrics, Xiamen University, Xiamen, China; ^3^ Fujian Provincial Key Laboratory of Ophthalmology and Visual Science, School of Medicine, Eye Institute of Xiamen University, Xiamen University, Xiamen, China; ^4^ Department of Clinical Pharmacy, The Third Hospital of Mianyang/Sichuan Mental Health Center, Mianyang, China; ^5^ Department of Pathology, Qilu Hospital, Shandong University, Jinan, China; ^6^ Technical Innovation Center for Utilization of Marine Biological Resources, Third Institute of Oceanography, Ministry of Natural Resources, Xiamen, China; ^7^ Key Laboratory of Functional and Clinical Translational Medicine, Xiamen Medical College, Fujian Province University, Xiamen, China; ^8^ CAS Key Laboratory of Design and Assembly of Functional Nanostructures and Fujian Provincial Key Laboratory of Nanomaterials, Fujian Institute of Research on the Structure of Matter, Chinese Academy of Sciences, Fujian, China; ^9^ Xiamen Institute of Rare-earth Materials, Haixi Institutes, Chinese Academy of Sciences, Fujian, China

**Keywords:** N-acylethanolamine acid amidase (NAAA), fatty acid ethanolamides (FAEs), anandamide (AEA), palmitoylethanolamide (PEA), analgesic tolerance

## Abstract

N-Acylethanolamine acid amidase (NAAA) is a lysosomal enzyme responsible for the hydrolysis of fatty acid ethanolamides (FAEs). However, the role of NAAA in FAEs metabolism and regulation of pain and inflammation remains mostly unknown. Here, we generated NAAA-deficient (NAAA^-/-^) mice using CRISPR-Cas9 technique, and found that deletion of NAAA increased PEA and AEA levels in bone marrow (BM) and macrophages, and elevated AEA levels in lungs. Unexpectedly, genetic blockade of NAAA caused moderately effective anti-inflammatory effects in lipopolysaccharides (LPS)-induced acute lung injury (ALI), and poor analgesic effects in carrageenan-induced hyperalgesia and sciatic nerve injury (SNI)-induced mechanical allodynia. These data contrasted with acute (single dose) or chronic NAAA inhibition by F96, which produced marked anti-inflammation and analgesia in these models. BM chimera experiments indicated that these phenotypes were associated with the absence of NAAA in non-BM cells, whereas deletion of NAAA in BM or BM-derived cells in rodent models resulted in potent analgesic and anti-inflammatory phenotypes. When combined, current study suggested that genetic blockade of NAAA regulated FAEs metabolism and inflammatory responses in a cell-specifical manner.

## Introduction

Fatty acid ethanolamides (FAEs) are endogenous lipids that participate in the modulation of many physiological functions (Piomelli and Sasso, 2014). Anandamide (AEA), oleoylethanolamide (OEA) and palmitoylethanolamide (PEA) are 3 of the most studied FAEs (Piomelli and Sasso, 2014). AEA is also known as the endocannabinoid, and has been identified as an agonist of cannabinoid receptors (CB1 and CB2) and transient receptor potential vanilloid type 1 (TRPV1) (Devane et al., 1992; Zygmunt et al., 1999). OEA, an agonist of peroxisome proliferator-activated receptor *α* (PPAR-*α*), TRPV1 and GPR119, mediates anorexigenic and anti-obesity effects (Fu et al., 2003; Petrosino et al., 2010). PEA has been known to generate anti-inflammatory and analgesic activity, modulated through PPAR-*α* (Fu et al., 2003; Lo Verme et al., 2005).

AEA, OEA and PEA are mainly metabolized by fatty acid amide hydrolase (FAAH) and N-acylethanolamine acid amidase (NAAA) (McKinney and Cravatt, 2005; Tsuboi et al., 2007a). FAAH is a membrane-bound serine hydrolase that highly distributed in liver and brain (McKinney and Cravatt, 2005), and has been identified as the major enzyme for the hydrolysis of AEA and OEA (Clapper et al., 2010; Ahn et al., 2011). Apart from FAAH, the degradation of AEA and OEA is attributed to NAAA (Tsuboi et al., 2005). NAAA is a cysteine hydrolase generally expressed in immune cells, e.g., monocytes and macrophages (Tsuboi et al., 2007b; Gorelik et al., 2018). In contrast to FAAH, NAAA is mainly located in lysosome and its bioactivity is optimal at pH of 4.5–5.0 (Tsuboi et al., 2005). Although NAAA is confirmed as the primary hydrolytic enzyme for hydrolysis of PEA, FAAH has been identified as another hydrolase for PEA (McKinney and Cravatt, 2005). The roles of FAAH on metabolism of FAEs have been well studied (Roques et al., 2012; Piomelli and Sasso, 2014; Carnevali et al., 2017). Both FAAH-deficient mice and pharmacological administration of FAAH inhibitors in animal result in significant increment of AEA (7∼15-fold), OEA (13-fold) and PEA (4∼8-fold) in various organs, e.g., brain and liver (Ahn et al., 2011; Li et al., 2012a). Contrary to FAAH, the role of NAAA in FAEs metabolism remains mostly unclear.

NAAA is believed to primarily control PEA levels and has been proposed as an alternative target for activation of PEA/PPAR*α* signaling (Tsuboi et al., 2005; Tai et al., 2012; Bottemanne et al., 2018). To date, many potent NAAA inhibitors have been developed, some of them were observed to elevate PEA levels and exhibit potent anti-inflammatory and analgesic effects in a range of rodent models of human disease (Alhouayek et al., 2015; Bonezzi et al., 2016; Alhouayek et al., 2017; Bottemanne et al., 2018; Sagheddu et al., 2019; Fotio et al., 2020; Piomelli et al., 2020; Sgroi et al., 2021). However, the impact of NAAA on AEA and OEA degradation has been poorly investigated. The genetic evidences are needed to further clarify the role of NAAA in FAEs metabolism and regulation of pain and inflammation. In the present study, we generated NAAA-deficient (NAAA^-/-^) mice using CRISPR-Cas9 technique, and measured FAEs levels in different tissues from wild-type (WT) and NAAA^-/-^ mice. We found that genetic deletion of NAAA regulates FAEs metabolism and causes analgesic and anti-inflammatory tolerance in a tissue-specifical manner. Current studies provided new insights in the role of NAAA in FAEs metabolism and inflammatory responses.

## Materials and Methods

### Chemicals

All reagents used in current study were obtained from Sinopharm (Shanghai, China), seeking the highest grade commercially available unless otherwise indicated. F96 was synthesized following our previously reported method (Ren et al., 2017).

### Animal

All animal experiments were performed in accordance with guide and care and use of laboratory animals from National Institutes of Health (NIH) and ARRIVE, and approved by the animal care and use committees of Xiamen University in China. C57BL/6 J mice were purchased from laboratory animal center of Xiamen university.

### NAAA^-/-^ Mice

NAAA^-/-^ mice were prepared by a clustered regularly interspaced short palindromic repeats (CRISPR)-Cas9 approach (Ran et al., 2013). Single-guide RNA (sgRNA) sequences targeting *naaa* exon1 (gene ID: 67111) were screened on the website of http://crispr.mit.edu, and synthesized by TsingKe Biological Technology (Hangzhou, China). After *in vitro* cleavage efficiency assay, one pair of sgRNAs for NAAA knockout were used, and the primer sequences were sgRNA1: 5’-GGT​CGG​AGA​AAT​TGT​GTC​GA-3’, sgRNA2: 5’-GCT​GCG​GAT​CTC​GTC​GGT​GA-3’.

NAAA sgRNA (20 μg) and Cas9 mRNA (10 μg) were then microinjected into the cytoplasm of collected fertilized eggs. After incubation for 24 h at 37°C, the 2-cell forms of the eggs were then transplanted to the ampulla of recipient pseudopregnancy ICR female mice. Genome DNA was extracted from the tail tip of F0 mice, followed by PCR amplification. After purification, PCR products were sequenced with Sanger sequencing and analyzed with the SnapGene 3.1.1 software. The male founder NAAA knockout mice (F0) were mated to naïve C57BL/6 mice to expand the colony, and NAAA^-/-^ mice were obtained from F2 heterozygous. RT-PCR, western-blot and enzyme activity measurements were performed to identify the NAAA^-/-^ mice.

### Real-Time Quantitative PCR

Total RNA was extracted from tissues with TRIzol (Invitrogen) and measured by spectrophotometer (Beckman coulter, Shanghai, China). cDNA was synthesized from 1 μg of total RNA using ReverTra Ace qPCR RT Kit (TOYOBO, Shanghai, China) following the manufacturer’s instructions. Realtime quantitative PCR was performed in a 7300 real-time PCR System (Applied Biosystems, CA, United States) using SYBR Premix Ex Taq GC (Takara, Dalian, China) (Li et al., 2012b). The amplification steps included denaturation at 95°C for 30 s (1 cycle), annealing at 56°C for 1 min (1 cycle), and extension at 72°C for 1 min (34 cycles). RNA levels were normalized using glyceraldehyde-3-phosphate dehydrogenase (GAPDH) as a reference gene. The primer sequences for mouse genes were as follows:

NAAA: 5’-TGC​CCT​TAC​AAT​CCT​CCT​GC-3’ (forward), 5’-TGC​CAT​CTA​GAT​CCC​TGA​CA-3’ (reverse).

IL1*β*: 5’-TCG​CTC​AGG​GTC​ACA​AGA​AA-3’ (forward), 5’-CAT​CAG​AGG​CAA​GGA​GGA​AAA​C-3’ (reverse).

TNF*α*: 5’-AGC​CCC​CAG​TCT​GTA​TCC​TT-3’ (forward), 5’-GGT​CAC​TGT​CCC​AGC​ATC​TT-3’ (reverse).

### Western-Blot

Protein isolation and western blots were performed as we described previously (Zhou et al., 2019). Antibodies against the following proteins were used: goat anti-mouse NAAA antibody (R&D, Cat#: AF4886, dilution 1:2000) and rabbit anti-mouse *β*-Actin (Abcam, Cat#: ab181602, dilution 1:2000). Bands were visualized with an electrochemiluminescence plus kit (Amersham Biosciences). Quantitative analyses were performed with Image J software, using *β*-Actin as the internal standard.

### Histology

After mice were killed, corresponding tissues were harvested and fixed in paraformaldehyde at 4°C, following by paraffin embedding and cutting into sections with a thickness of 5 μm. H&E staining and immunochemistry were performed on paraffin embedded tissues samples using previously described methods (Wu et al., 2019; Zhou et al., 2019). Primary rabbit anti-mouse NAAA antibody (R&D, Cat#: AF4886, dilution 1:400) were used. Images were taken with an Olympus microscope at 10 × magnification. To confirm the antibody binding specificity, some sections were also incubated with primary or secondary antibody only.

The H&E-stained image was analyzed and scored using a literature procedure (Wu et al., 2019). The H&E-stained sections were assessed in a semiquantitative fashion according to already published criteria: The evaluations were recorded as the perivascular edema (P1), the perivascular immune cell recruitment (P2), goblet cells in bronchioles (P3), and macrophages in alveolar spaces (P4). P1 was evaluated using the criteria of spaces surrounded veins: 0 = no change; 1 = 0–25% of the perivascular spaces; 2 = 25%–75%; 3 = > 75%. P2 was evaluated using the criteria of immune cells surrounded veins: 0 = absent; 1 = < 3 cell thick; 2 = 3–5 cells thick; 3 = > 5 cells thick. P3 was evaluated using the criteria of goblet cells present in two bronchiolar profiles: 0 = absent; 1 = < 10 cells; 2 = > 10 cells. P4 was evaluated using the criteria of eosinophilic macrophages present in two alveolar spaces: 0 = absent; 1 = < 10 cells; 2 = > 10 cells. The total H&E score was expressed as the sum of the individual scores: H score = P1 + P2 + P3 + P4. All of the H&E-stained sections were scored blindly and independently by at least two investigators.

### PEA Hydrolytic Activities

The lysosomal fraction was prepared using a previously described method. Lungs, spleen and brain were immediately dissected after mice were sacrificed by CO_2_ inhalation. Tissues from three mice were homogenized together in 50 mM Tris-HCl buffer (pH 7.4, 0.32 M sucrose, 2.5 ml for lungs or brain, 2 ml for spleen) for 5 min at 4°C. Samples were centrifuged at 1000 × g for 10 min at 4°C, and the resulting supernatants were then subjected to three freeze–thaw cycles at −80°C, followed by centrifugation again at 12,000 × g for 30 min at 4°C. The supernatant was finally centrifuged at 100,000 × g for 60 min at 4°C. The supernatant was collected, protein concentration was measured by BCA protein assay kit and samples were stored at −80°C until use. PEA hydrolytic activities of related samples were tested using methods as previously described by our group (Li et al., 2018).

### Lipid Extraction and LC-MS/MS Analysis

Lipids were extracted and analyzed using previously described method (Yang et al., 2015; Li et al., 2018).

### Primary Macrophages Culture

Primary macrophages were isolated as previously described (Li et al., 2018). Peritoneal cells of WT and NAAA-null mice (8–14 weeks) were isolated by lavage with 4 ml PBS and 1 × 10^6^ cells were incubated in 12-well plates at 37°C for 3 h in DMEM supplemented with 10% FBS and 2 mM glutamine. Medium was then discarded and non-adherent cells were removed by washing 3 times with PBS. Remaining cells were defined as peritoneal macrophages. Cells were harvested and homogenized in 2 ml methanol-water (1:1) containing internal standard C17:1 FAE, and then extracted with chloroform. The organic phase was concentrated under a nitrogen atmosphere and reconstituted in methanol for LC/MS/MS analyses.

### Carrageenan-Induced Local Inflammation

Briefly, male C57BL/6J (WT) mice or NAAA^-/-^ mice (20–25 g) were grouped (*n* = 5), and injected λ-carrageenan (1% (w/v) in saline, 50 μL) into the left hind paw. WIN55,212-2 (2 mg/kg), JWH-133 (2 mg/kg), GW6471 (2 mg/kg), F96 (30 mg/kg) or their vehicle were intravenously (*i.v.*) injected 2 h after carrageenan administration. In separate groups of WT mice, F96 (30 mg/kg) was intravenously injected once daily for 7 days, final dose was administered 2 h after carrageenan injection. Pain behavior was performed 6 h after carrageenan injection as we described previously (Zhou et al., 2019).

### Sciatic Nerve Injury (SNI)-Induced Neuropathic Pain

Briefly, male C57BL/6J (WT) mice or NAAA^-/-^ mice (20–25 g) were grouped (n = 5), anesthetized with chloral hydrate (400 mg/kg). We performed the SNI model with the same surgical procedure previously described by our group (Yang et al., 2015). F96 (30 mg/kg, *i.v.*, once daily) or its vehicle was administered starting from the day of SNI surgery. Pain behavior was performed in day 3 and 7 at 2 h after F96 injection.

### Lipopolysaccharides (LPS)-Induced Acute Lung Injury (ALI) Model

Briefly, male C57BL/6 J (WT) mice or NAAA^-/-^ mice (20–25 g) were grouped (*n* = 5), anesthetized with chloral hydrate (400 mg/kg) and instilled intratracheally with LPS (7 mg/kg) in PBS (40–50 μL). In separate groups of WT mice, mice were treated with F96 (30 mg/kg, *i.v*. 6 h after LPS administration). All animals were sacrificed 24 h after LPS administration. The bronchoalveolar lavage fluid (BALF) was obtained by intratracheal intubation. Lungs were infused with 0.6 ml of physiological saline for three times to collect a total of 1.5 ml of BALF. The BALF sample was centrifuged at 500 × g for 15 min at 4°C, and the supernatants were measured by BCA protein assay kit. The lung was then dissected for PCR and histopathological studies.

### Transplantation of Bone Marrow (BM)

Transplantation of BM was performed using a previous reported method (Singh et al., 2016; Wang et al., 2018). Briefly, C57BL/6J (WT) or NAAA^-/-^ mice (25–28 g) were irradiated (700 Rad × 2), and received 5 × 10^6^ BM cells isolated from either WT or NAAA^-/-^ donor mice by intravenous injection. The transplanted mice were used for experiments after 8 weeks. Chimeric mice were verified by flow cytometry at day 0, 2 and 55 after transplantation. Irradiated WT mice transplanted with WT BM, irradiated NAAA^-/-^ mice transplanted with NAAA^−/-^ BM, irradiated NAAA^−/-^ mice transplanted with WT BM [NAAA^−/-^ (r) + WT(d)] and irradiated WT mice transplanted with NAAA^−/-^ BM [WT (r) + NAAA^−/-^ (d)] were grouped (*n* = 5), and were subjected to the LPS-induced ALI model or carrageenan-induced local inflammation model.

### Statistical Analysis

Randomization was used to assign mice to different experimental groups, to collect and process data. All histology samples were scored blindly and independently by at least two investigators. Results were showed as the mean ± SEM. They were analyzed by one-way ANOVA with Dunnett’s post hoc multiple comparison tests. For all ANOVA, post hoc tests were run only if F achieved *p* < 0.05 and there was no significant variance inhomogeneity. *p* < 0.05 was considered statistically significant.

## Results

### CRISPR/Cas9-Mediated Deletion of Naaa in C57BL/6 Mice

**FIGURE 1:**
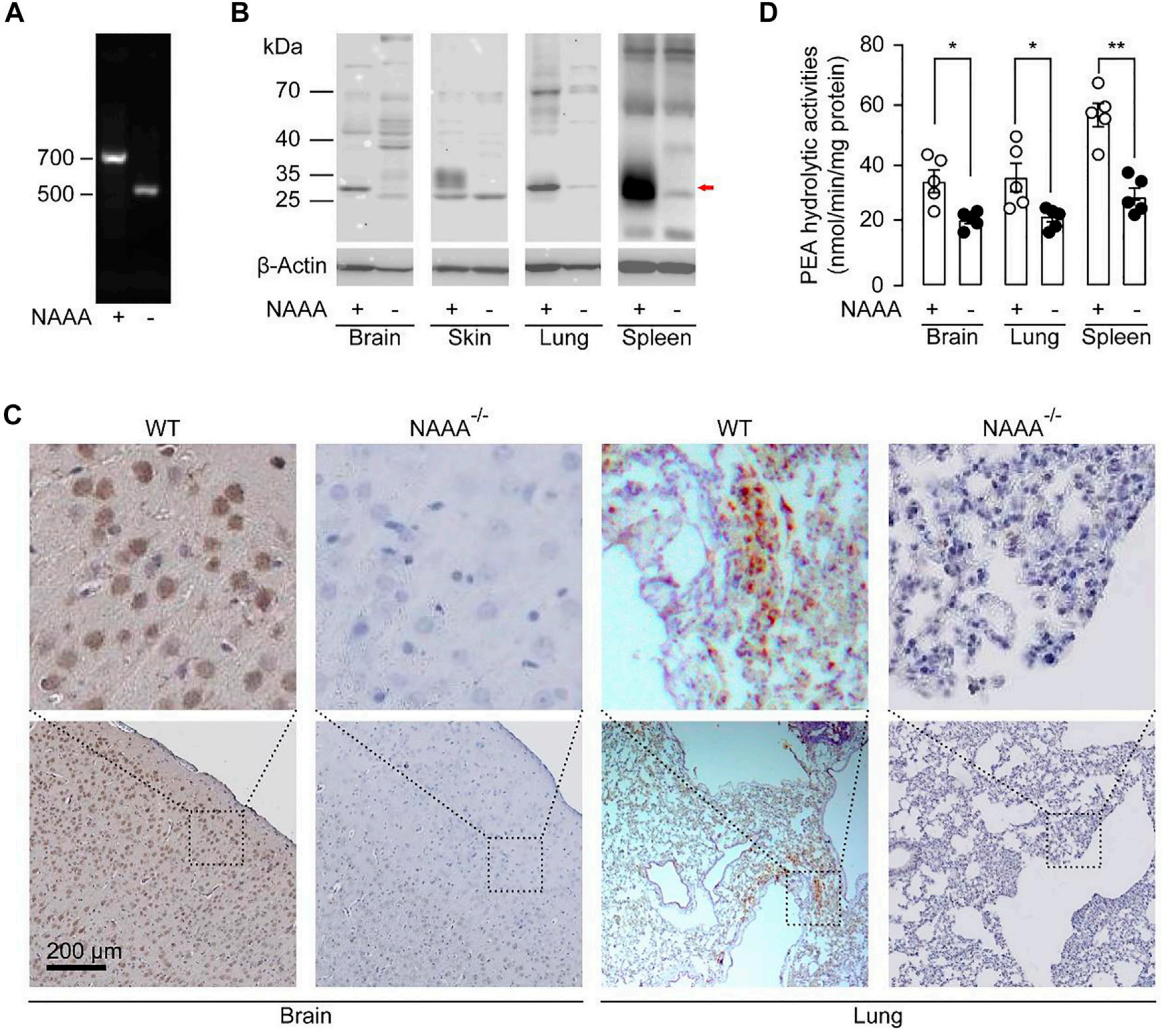
Characterization of NAAA^-/-^ mice. (**A)** Tail genomic analysis of WT and NAAA^−/-^ mouse by PCR. **(B)** Representative western-blot bands of NAAA abundances in the spleen, lungs, brain, and skin from WT and NAAA^-/-^ mice. **(C)** Representative immunohistochemical analyses of NAAA in the brain and lung sections from WT and NAAA^−/-^ mice. **(D)** PEA hydrolytic activities were tested in the lysosomal fraction of brain, lungs and spleen homogenates from WT and NAAA^−/-^ mice, at pH 4.5. Data are expressed as mean ± SEM (*n* = 5). *, *p* < 0.05; **, *p* < 0.01 by *t* test.

First, we generated the NAAA-deficient (NAAA^-/-^) mice using CRISPR-Cas9 technique (Ran et al., 2013). Two different sgRNAs that bind to exon 1 within the *naaa* gene were used to create a homozygous knockout in C57BL/6 mice. The obtained NAAA^-/-^ mice were verified by PCR, western blot, immunochemistry and PEA hydrolytic activity analysis. The results showed that the predicted sizes of the PCR product from the wild-type (WT) and NAAA^-/-^ mice are 744 and 488 bp, respectively (Figure 1A). Sanger sequencing of the PCR products further confirmed a homozygous 258 bp deletion and 2 bp insertion in exon 1 (Supplementary Figure S1). Western blot showed that 29 kDa (*β*-subunit) bands were hardly detectable in brain, lungs, skin and spleen samples from NAAA^-/-^ mice (Figure 1B). Immunohistochemical analysis suggested that the NAAA-positive cells disappeared in the brain and lungs of NAAA^-/-^ mice (Figure 1C). Additionally, the PEA hydrolytic activities at pH 4.5 in spleen, lungs and brain from NAAA^-/-^ mice were reduced nearly 50%, when compared with WT group (Figure 1D). These data indicated that Cas9 protein successfully cleaved *naaa* gene in NAAA^-/-^ mice.

We also tested whether deletion of NAAA changed the expression of other synthetic and degradative enzymes of FAEs. N-acyl-phosphatidylethanolamine phospholipase D (NAPE-PLD) is the major synthetic enzyme of FAEs, while FAAH could degrade AEA. Western blotting showed that there was no significant difference in protein levels of these enzymes in lungs of NAAA^-/-^ mice, compared to WT mice (Data not shown). Additionally, NAAA^-/-^ homozygous mice were viable, fertile, and largely indistinguishable from WT littermates.

### Genetic Inactivation of NAAA Impairs FAEs Levels *in vivo*

**FIGURE 2:**
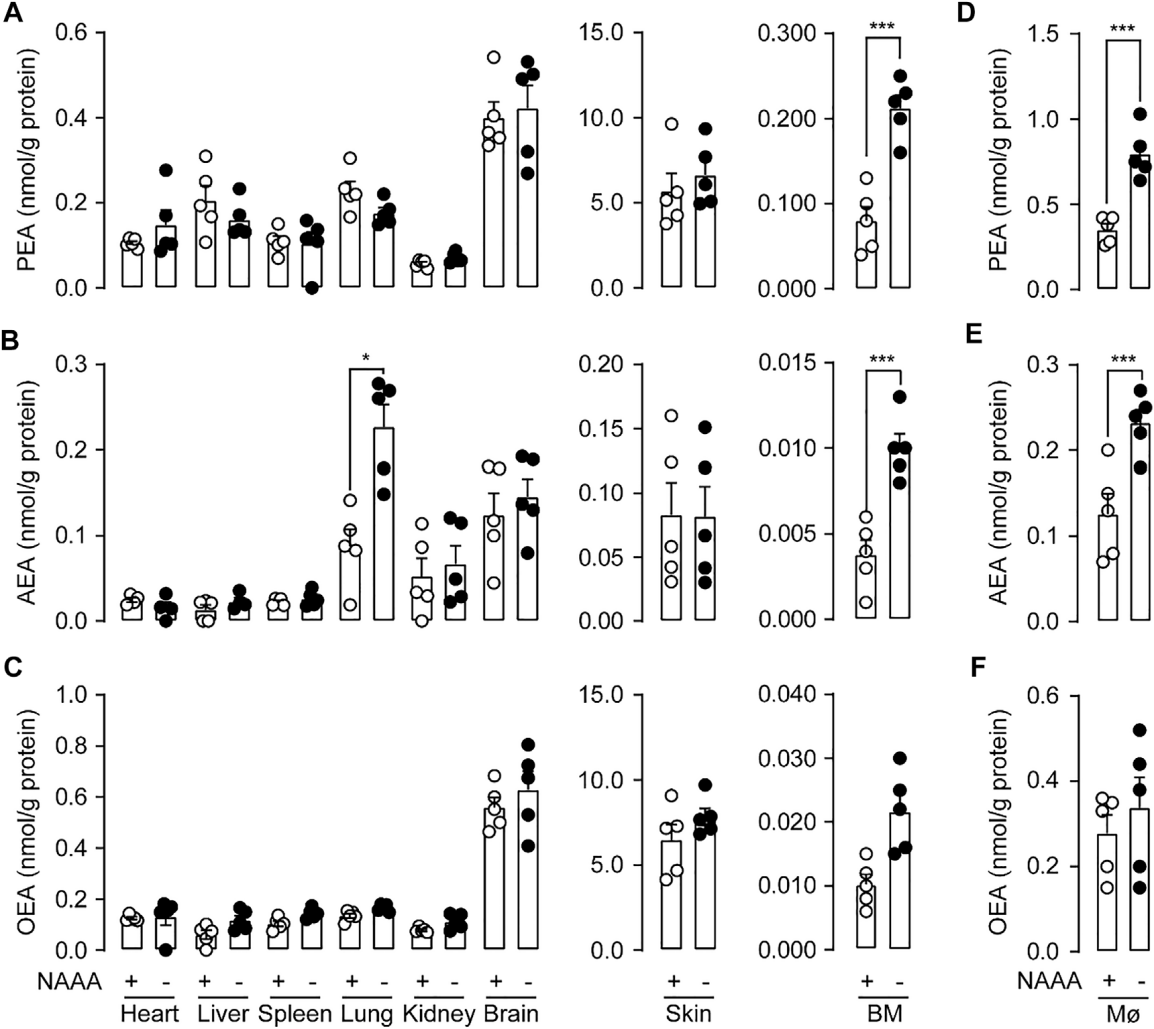
Genetic inactivation of NAAA impairs FAEs levels ***in vivo***. PEA **(A,D)**, AEA **(B,E)** and OEA **(C,F)** levels in various organs and bone marrow (BM) and primary macrophages (MØ) in WT and NAAA^-/-^ mice. Data are expressed as mean ± SEM (*n* = 5). ns., *p* > 0.05; *, *p* < 0.05, ***, *p* < 0.001 by *t* test.

Next, we asked whether genetic inactivation of NAAA affected FAEs levels in mice. Although NAAA is responsible for the degradation of PEA, NAAA blockade had poor effects on PEA levels in heart, liver, spleen, lungs, kidney, skin, and brain in mice (Figure 2A). The only exception was in bone marrow (BM), where NAAA blockade increased PEA levels by nearly 2.5-fold (Figure 2A). Similarly, no differences were observed in AEA contents in heart, liver, spleen and kidney from NAAA^-/-^ and WT mice (Figure 2B). Genetic inactivation of NAAA only increased AEA levels in lungs (2.6-fold) and BM (3-fold) (Figure 2B). Additionally, NAAA blockade had no effects on OEA levels in all tissues (Figure 2C). NAAA has been demonstrated to express in myeloid-derived immune cells, e.g., monocytes and macrophages (Tsuboi et al., 2005), we then tested whether genetic knockout of NAAA impairs FAEs metabolisms in these cells. Primary macrophages extracted from NAAA^-/-^ mice showed nearly 2-fold higher PEA and AEA levels compared to WT group (Figures 2D,E). Additionally, no significant difference was observed in OEA levels between primary macrophages from WT and NAAA^-/-^ mice. (Figure 2F). When combined, these data indicated that NAAA contributed significantly to the degradation of PEA in BM and macrophages, and the metabolism of AEA in lungs, BM and macrophages.

**FIGURE 3:**
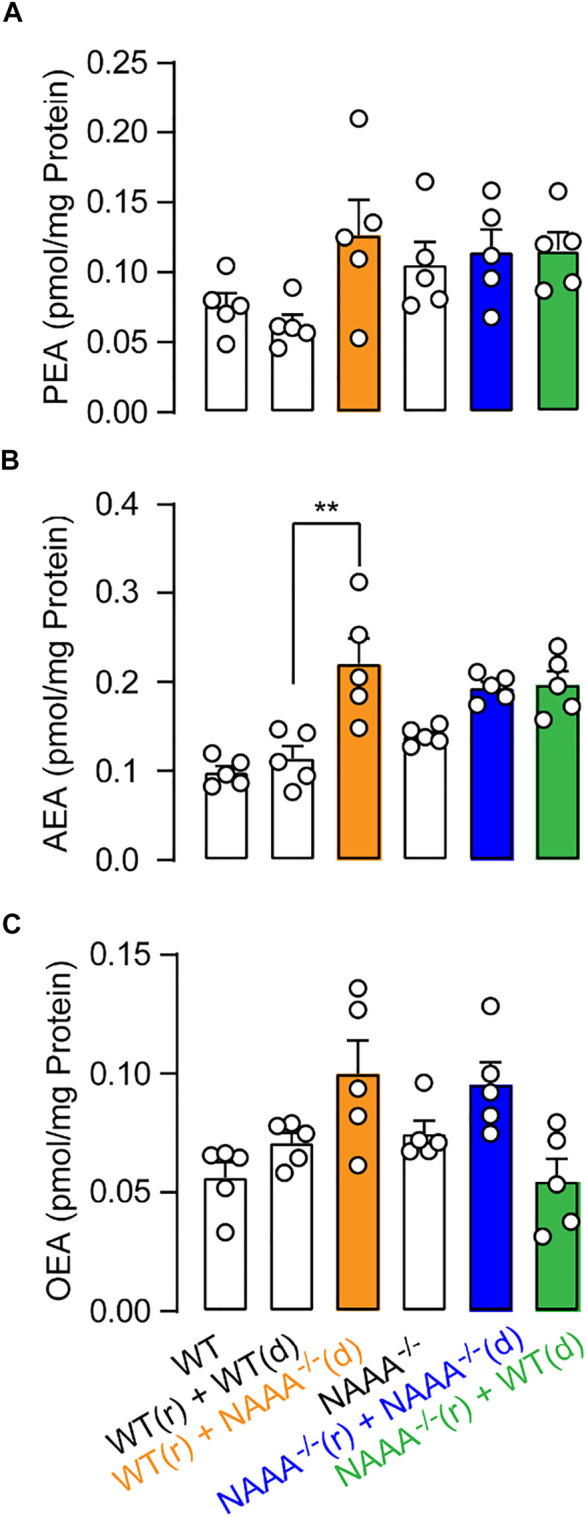
Genetic inactivation of NAAA in BM-derived cells increases PEA and AEA levels in lungs. BM isolated from WT (d) or NAAA^-/-^ (d) mice was individually transferred into lethally irradiated WT (r) or NAAA^-/-^ (r) mice. Chimeras were used for HPLC-MS studies 8 weeks after initial reconstitution. **(A)** PEA, **(B)** AEA and **(C)** OEA levels in lungs in chimera mice. Data are expressed as mean ± SEM (*n* = 5). *, *p* < 0.05; **, *p* < 0.01; ***, *p* < 0.001 by *t* test.

To examined what kinds of cells expressed NAAA contributed to FAEs metabolism in lungs, we transferred BM from WT or NAAA^-/-^ mice into lethally irradiated WT or NAAA^-/-^ mice, respectively. We observed that deletion of NAAA in BM or BM-derived cells increased AEA levels (nearly 2-fold) in lungs (Figure 3B), whereas the chimeric mice that lacked NAAA in non-BM cells showed no increase in AEA levels, suggesting that NAAA in BM cells regulates AEA metabolism in lungs. In addition, deletion of NAAA in neither BM nor non-BM cells do not affect PEA and OEA levels in lungs (Figures 3A,C). These data suggested that NAAA regulates pulmonary AEA metabolism largely through targeting BM or BM-derived cells, most likely BM-derived immune cells.

### Genetic Inactivation of NAAA Causes the Anti-inflammatory and Analgesic Tolerance

**FIGURE 4:**
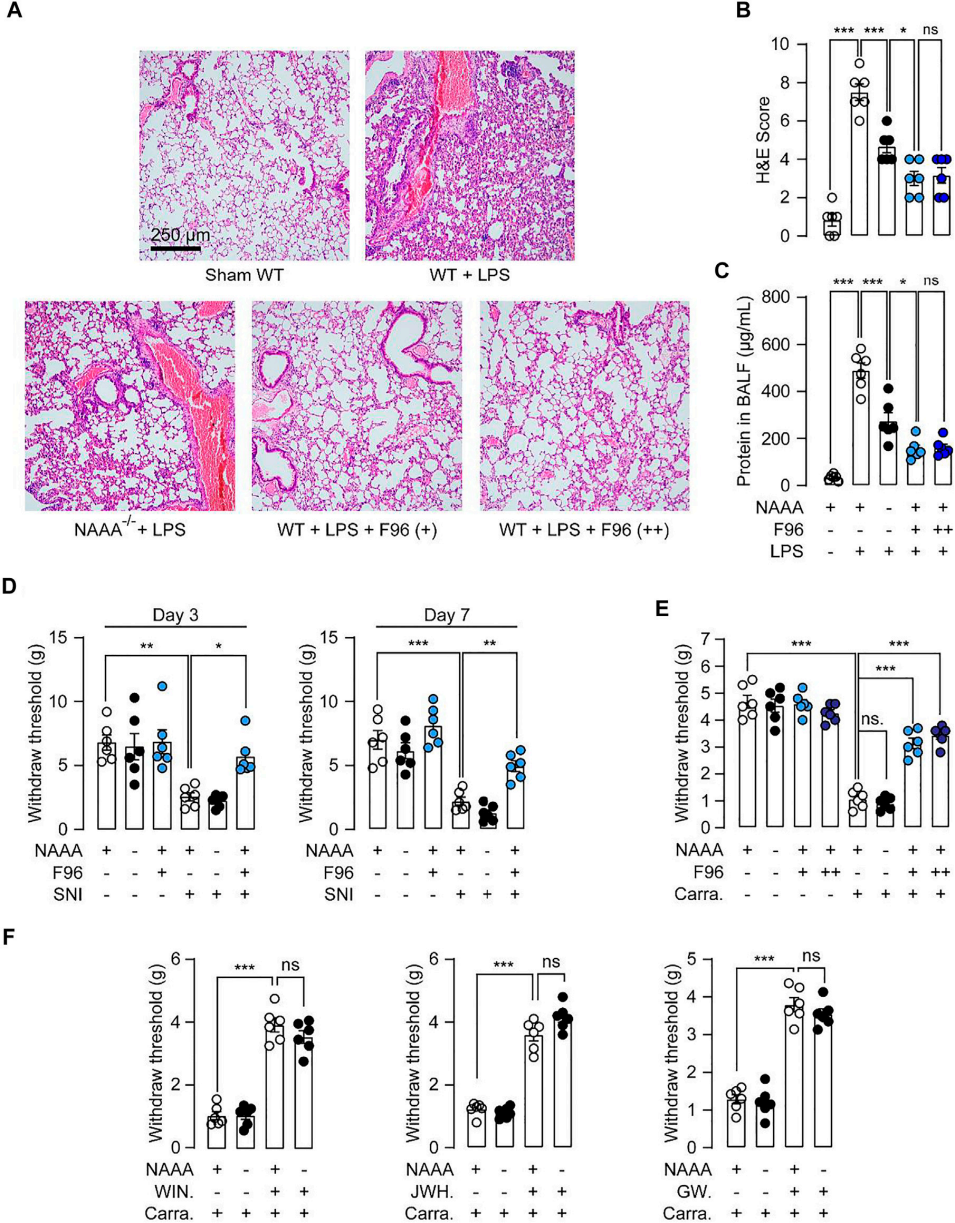
Genetic inactivation of NAAA causes analgesic and anti-inflammatory tolerance. **(A,B)** WT and NAAA^−/-^ mice were instilled intratracheally with saline or LPS, following by treatment with vehicle or F96 (30 mg/kg, +, *i.v*., 6 h after LPS administration). In separate groups of WT mice, F96 (30 mg/kg, ++) was intravenously injected once daily for 7 days, final dose was administered 6 h after LPS administration. H&E staining lung sections of sham and ALI mice sacrificed 24 h after LPS administration. **(C)** Protein concentrations in BALF in sham and ALI mice. **(D)** WT and NAAA^-/-^ mice were received sham or SNI operation, following by treatment with vehicle or F96 (30 mg/kg, *i.v*., once daily for 7 days), and measured hyperalgesia at day 3 and 7 post surgery. (**E,F**) WT and NAAA^−/-^ mice were intra-plantar injected with 1% *λ*-carrageenan or its saline vehicle. WIN55,212-2 (2 mg/kg), JWH-133 (2 mg/kg), GW6471 (2 mg/kg), F96 (30 mg/kg, +) or vehicle were intravenously (*i.v.*) injected 2 h after carrageenan administration. In separate groups of WT mice, F96 (30 mg/kg, ++) was intravenously injected once daily for 7 days, final dose was administered 2 h after carrageenan injection. Hyperalgesia was measured at 6 h after carrageenan injection. Data are expressed as mean ± SEM (*n* = 5–6). *, *p* < 0.05; **, *p* < 0.01; ***, *p* < 0.001 by one-way ANOVA.

Subsequently, we asked whether genetic inactivation of NAAA could reduce inflammation and pain. We first assessed whether genetic inactivation of NAAA might be beneficial in LPS-induced acute lung injury (ALI) model. Intratracheal instillation of LPS induced severe lung injury, including hemorrhage, leukocyte infiltration and alveolar edema in WT mice (Figures 4A,B). By contrast, F96-treated mice and NAAA^-/-^ mice displayed significantly less edema and fewer infiltrated immune cells than WT mice (Figures 4A,B). Accumulation of protein-rich fluid in alveolar spaces is an important symptom of lung injury. Both genetic inactivation and pharmacological administration of NAAA inhibitor F96 in mice significantly reduced alveolar protein levels in BALF (Figure 4C). Interestingly, F96-treated mice showed thinner alveolar walls with few neutrophils, and lower protein level in BALF than NAAA^-/-^ mice (Figures 4A–C). These results showed that genetic deletion of NAAA weakens the anti-inflammatory effects of NAAA inhibitor.

Next, we further studied the behavioral consequences of genetic blockade of NAAA using the SNI-induced neuropathic pain model and carrageenan-induced hyperalgesia model. SNI surgery induced significant decrease of mechanical withdraw thresholds in WT mice, repeated administration of F96 greatly blocked SNI-induced neuropathic pain in WT mice on day 3 and 7 post-surgery (Figure 4D). However, NAAA^-/-^ mice displayed equivalent paw withdraw thresholds as WT mice (Figure 4D). Similarly, intra-plantar injection of carrageenan into one of the hind paws in WT mice resulted in the development of paw inflammatory hyperalgesia (Figure 4E). Although NAAA inhibitor F96 significantly reduced pain behavior, NAAA^-/-^ mice showed similar pain responses as control mice (Figure 4E). These results suggested that the analgesic effects produced by NAAA inhibition are lost following genetic knockout of this enzyme.

Genetic inactivation of endocannabinoid hydrolase, such as MAGL, or chronic treatment of MAGL inhibitor, causes analgesic tolerance and functional antagonism of the endocannabinoid system (Schlosburg et al., 2010). To investigated whether this form of tolerance was associated with chronic blockade of NAAA activity, we assessed the inflammation and pain responses of mice treated chronically for six consecutive days with the NAAA inhibitor F96. Mice chronically treated with F96 showed similar inflammation and pain responses as treated with F96 acutely (single dose) (Figures 4A–C,E), indicating that chronic blockade of NAAA activity cannot lead to the development of tolerance. We also investigated whether genetic inactivation of NAAA results in receptor desensitization. We assessed the behavioral effects of receptor agonists on carrageenan-induced hyperalgesia in NAAA^-/-^ mice. As shown in Figure 4F, no differences were observed for pain behavior in WT and NAAA^-/-^ mice after treatment with CB1 agonist WIN55,212-2, CB2 agonist JWH-133 or PPAR*α* agonist GW7647. These data suggested that genetic inactivation of NAAA does not cause functional antagonism of the PPAR*α* and endocannabinoid system.

### Genetic Inactivation of NAAA in Non-BM-derived Cells Causes the Anti-inflammatory and Analgesic Tolerance

**FIGURE 5:**
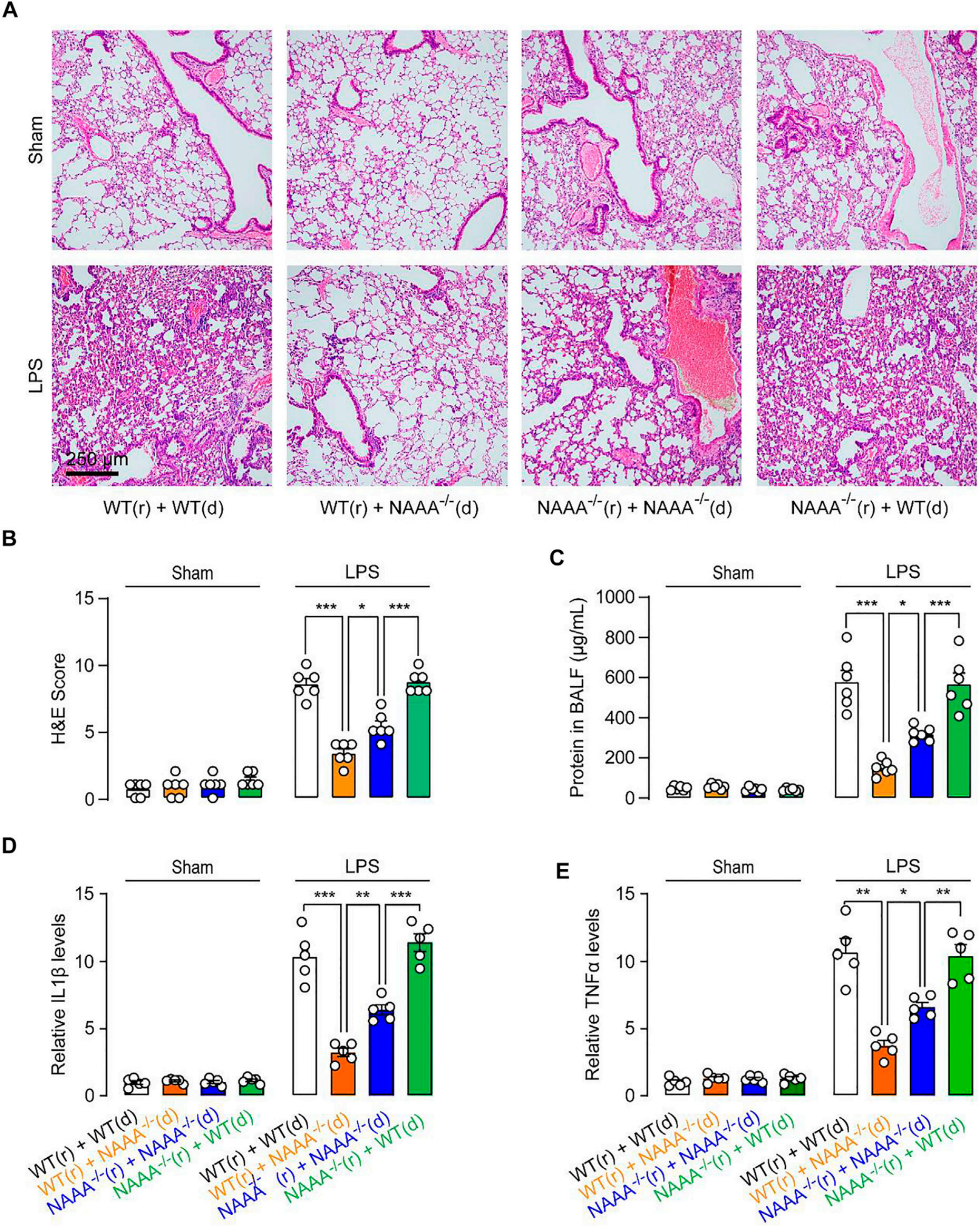
Genetic blockade of NAAA in non-BM cells cause anti-inflammatory tolerance. BM isolated from WT (d) or NAAA^−/-^ (d) mice was individually transferred into lethally irradiated WT (r) or NAAA^−/-^ (r) mice. Chimeras were used for studies 8 weeks after initial reconstitution. Chimeric mice were instilled intratracheally with saline or LPS, following by treatment with vehicle or F96 (30 mg/kg, *i.v*., 6 h after LPS administration). **(A,B)** H&E staining lung sections of chimeras sacrificed 24 h after LPS administration. **(C)** Protein concentrations in BALF in chimeras. **(D)** IL1*β* and **(E)** TNF*α* mRNA levels in lung tissues. Data are expressed as mean ± SEM (*n* = 5–6). *, *p* < 0.05; ***, *p* < 0.001 by one-way ANOVA.

To determine what kind of cell drives the anti-inflammatory and analgesic tolerance, we tested chimeric mice in LPS-induced ALI model. Transferring BM cells from WT mice into WT or NAAA^-/-^ mice caused the recipient mice to develop pulmonary inflammatory phenotypes (Figures 5A–E). Transferring BM cells from NAAA^-/-^ mice into WT or NAAA^−/-^ mice blocked the ALI development (Figures 5A–E). These results indicated that the NAAA expressed in BM or BM-derived cells plays a dominant role in inflammatory responses. Notably, transferring BM cells from NAAA^-/-^ mice into WT mice produced more potent anti-inflammatory effects than transferring into NAAA^-/-^ mice, indicating that genetic inactivation of NAAA in non-BM cells causes anti-inflammatory tolerance.

**FIGURE 6:**
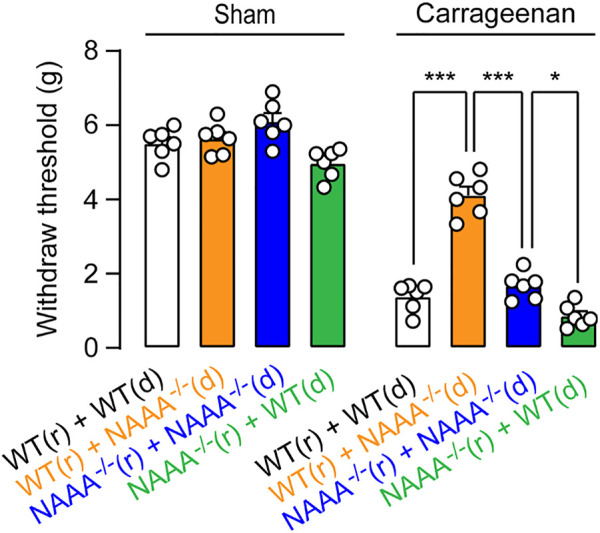
Genetic blockade of NAAA in non-BM cells cause analgesic tolerance. BM isolated from WT (d) or NAAA^-/-^ (d) mice was individually transferred into lethally irradiated WT (r) or NAAA^-/-^ (r) mice. Chimeras were used for behavior studies 8 weeks after initial reconstitution. Chimeric mice were intra-plantar injected with 1% *λ*-carrageenan or saline vehicle. Hyperalgesia was measured at 6 h after carrageenan or saline vehicle injection. Data are expressed as mean ± SEM (*n* = 5). *, *p* < 0.05; ***, *p* < 0.001 by one-way ANOVA.

A similar outcome was also observed in carrageenan-induced inflammatory pain. Transferring BM cells from WT mice into WT or NAAA^−/-^ mice caused the recipient mice to develop pain responses (Figure 6). Transferring BM cells from NAAA^−/-^ mice into WT or NAAA^-/-^ mice produced analgesia (Figure 6). Moreover, transferring BM cells from NAAA^-/-^ mice into WT mice produced more potent analgesic effects than transferring into NAAA^-/-^ mice (Figure 6). When combined, these results suggested that deletion of NAAA in BM or BM-derived cells reduce inflammatory and pain responses, while deletion of NAAA in non-BM cells caused analgesic and anti-inflammatory tolerance.

## Discussion

Over the last decades, increasing endogenous FAEs by inhibition of degrading enzymes has been suggested as a potential approach to treat inflammation, pain and other related disease (Bandiera et al., 2014). NAAA has been demonstrated to be an important hydrolase responsible for PEA degradation in immune cells, but the role of NAAA in FAEs metabolism and regulation of pain and inflammation remains unclear (Ueda et al., 2010). In the present work, we showed that genetic blockade of NAAA regulates FAEs metabolism and caused analgesic and anti-inflammatory tolerance in a tissue-specifical manner.

In the present work, we generated NAAA^-/-^ mice using the CRISPR-Cas9 technique. Our studies showed that NAAA^-/-^ homozygous mice were largely indistinguishable from WT littermates in general appearance and overt behavior, indicating NAAA can be regarded as a safe target for therapeutic intervention. NAAA is previously believed to primarily control PEA levels (Tsuboi et al., 2005; Tai et al., 2012; Bottemanne et al., 2018). However, our data suggested that genetic knockout of NAAA did not cause obvious change in the PEA levels in most of tissues (Figures 2A–C), suggesting its limited role in the PEA degradation under physiological condition. Both FAAH and NAAA are capable of hydrolyzing PEA, and blockade of FAAH in animal leads to significant increment of PEA (4∼8-fold) (Clapper et al., 2010). These data seem to suggest that the degradation of PEA in animal tissues may be attributed mostly by FAAH but not by NAAA. On the other hand, NAAA is abundant in immune cells and Carlos Solorzano et. al. showed that NAAA inhibitor (S)-OOPP but not FAAH inhibitor URB597 increased PEA level in leukocytes (Solorzano et al., 2009). Silvia Pontis et. al. found that overexpression of NAAA in monocyte-derived cells decreased PEA levels in tissues (Pontis et al., 2020). Consistent with these studies, we found that lack of NAAA in mice increased PEA levels of BM and macrophages (Figures 2D–F), indicating that NAAA but not FAAH might play a major role in hydrolyzing PEA in these immune cells.

Although NAAA do not play a dominant role on PEA metabolism in most of tissues under physiological condition, NAAA inhibitors have been reported to increase PEA levels in various disease models (Sasso et al., 2013; Li et al., 2018; Sagheddu et al., 2019), indicating that NAAA is implicated in the regulation of PEA levels under pathological conditions. This state-dependent metabolic property of NAAA may be partially explained by the redistribution of NAAA-contained cells in pathological tissues. During pathological state, e.g., traumatic brain injury, lung injury and colitis, NAAA abundant immune cells recruits to the injured tissues, enhancing the contribution of NAAA in PEA degradation (Alhouayek et al., 2015; Wu et al., 2019; Li et al., 2021). Therefore, NAAA inhibition shows poor effects on PEA levels under physiological condition but increases their content in disease models.

In the past decade, multiple potent NAAA inhibitors have been developed and shown to exhibit profound analgesic and anti-inflammatory effects in rodent models (Piomelli et al., 2020). In the current study, using NAAA^-/-^ mice, we found that genetic deletion of NAAA produced poor analgesic effects in carrageenan-induced hyperalgesia and SNI-induced mechanical allodynia (Figure 4). Additionally, although NAAA deletion significantly diminished the LPS-induced inflammatory response in lungs, the protective effects of NAAA inhibition by F96 were greater than NAAA insufficiency (Figures 4A–C). These data suggest that genetic deletion of NAAA causes analgesic and anti-inflammatory tolerance.

The precise reason for this phenotype is unclear. One possibility is the existence of compensatory mechanisms in the NAAA-null mice that counteract the pharmacological effects of NAAA inhibition. These compensatory mechanisms may occur in non-BM cells, including neurons, oligodendrocytes and alveolar epithelial cells (Pontis et al., 2020; Li et al., 2021; Fotio et al., 2021). Deletion of NAAA in BM or BM-derived cells produced profound analgesic and anti-inflammatory effects in mice, whereas the chimeric mice that lacked NAAA in non-BM cells, but not in BM cells, exhibited more inflammation and pain responses than NAAA^-/-^ mice (Figures 5, 6), supporting our hypothesis. Further studies are warranted to investigate the role of NAAA in non-BM cells. A second possibility is that chronic NAAA blockade desensitizes PPAR*α* or CB receptors desensitization in mice. There are many examples of functional antagonism of receptor systems following chronic blockade of a metabolic enzyme. For instance, chronic blockade of MAGL causes analgesic tolerance due to CB1 receptor desensitization (Schlosburg et al., 2010). Genetic deletion of acetylcholinesterase reduced nicotinic receptors activity mice (Sun et al., 2007). However, NAAA insufficiency did not affect the analgesic effects of PPAR*α* and CB receptor agonists, while repeated treatments with high doses of NAAA inhibitor produce analgesic effects, without caused of tolerance in pain behavior in mice (Figures 4D–F), indicating that the second mechanism do not play a dominant role. Additionally, F96 is very selective for NAAA over FAAH (155-fold), MAGL (>370-fold), CBs (>370-fold) and PPAR*α* (>370-fold) (Yang et al., 2015). However, we cannot exclude the possibility that F96 may interact with targets other than NAAA, producing more potent anti-inflammatory and analgesic effects than the genetic blockade of NAAA. Further studies are needed to confirm the specificities of F96 for NAAA.

Our results using the NAAA-null mice differ from those published by Fotio et al., who showed that genetic NAAA deletion blocked the chronic constriction injury (CCI)-induced thermal and mechanical hypersensitivity in mice (Fotio et al., 2021). We cannot explain the discrepancies between our findings and those of Fotio et al. The differences in the generation method of NAAA^−/-^ mice (i.e., CRISPR-Cas9 in this manuscript vs. loxP-Cre system by Fotio et al.) are one possible explanation (Pontis et al., 2020).

## Conclusion

Together, our results reveal a previously unexplored role for NAAA in FAEs metabolism and pain responses. Our data showed that NAAA regulates PEA and AEA levels in bone marrow (BM) and macrophages, and affects AEA levels in lungs. Deletion of NAAA in BM cells produces anti-inflammatory effects, while genetic blockade of NAAA in non-BM cells causes analgesic and anti-inflammatory tolerance.

## Data Availability

The original contributions presented in the study are included in the article/Supplementary Material, further inquiries can be directed to the corresponding authors.
