# Supplementary material for: Genetic Blockade of NAAA Cell-specifically Regulates Fatty Acid Ethanolamides (FAEs) Metabolism and Inflammatory Responses
Source: Front Pharmacol. 2022 Jan 7;12:817603. doi: 10.3389/fphar.2021.817603 (PMC8777083; doi:10.3389/fphar.2021.817603)
Supplement: Supplementary file 1 [file DataSheet1.DOCX]

Genetic blockade of NAAA impairs fatty acid ethanolamides (FAEs) metabolism and causes anti-inflammatory and analgesic tolerance

Xiaohua Xie, Yitian Li, Sengnan Xu, Pan Zhou, Longhe Yang, Yan Qiu, Yungang Yang and Yuhang Li


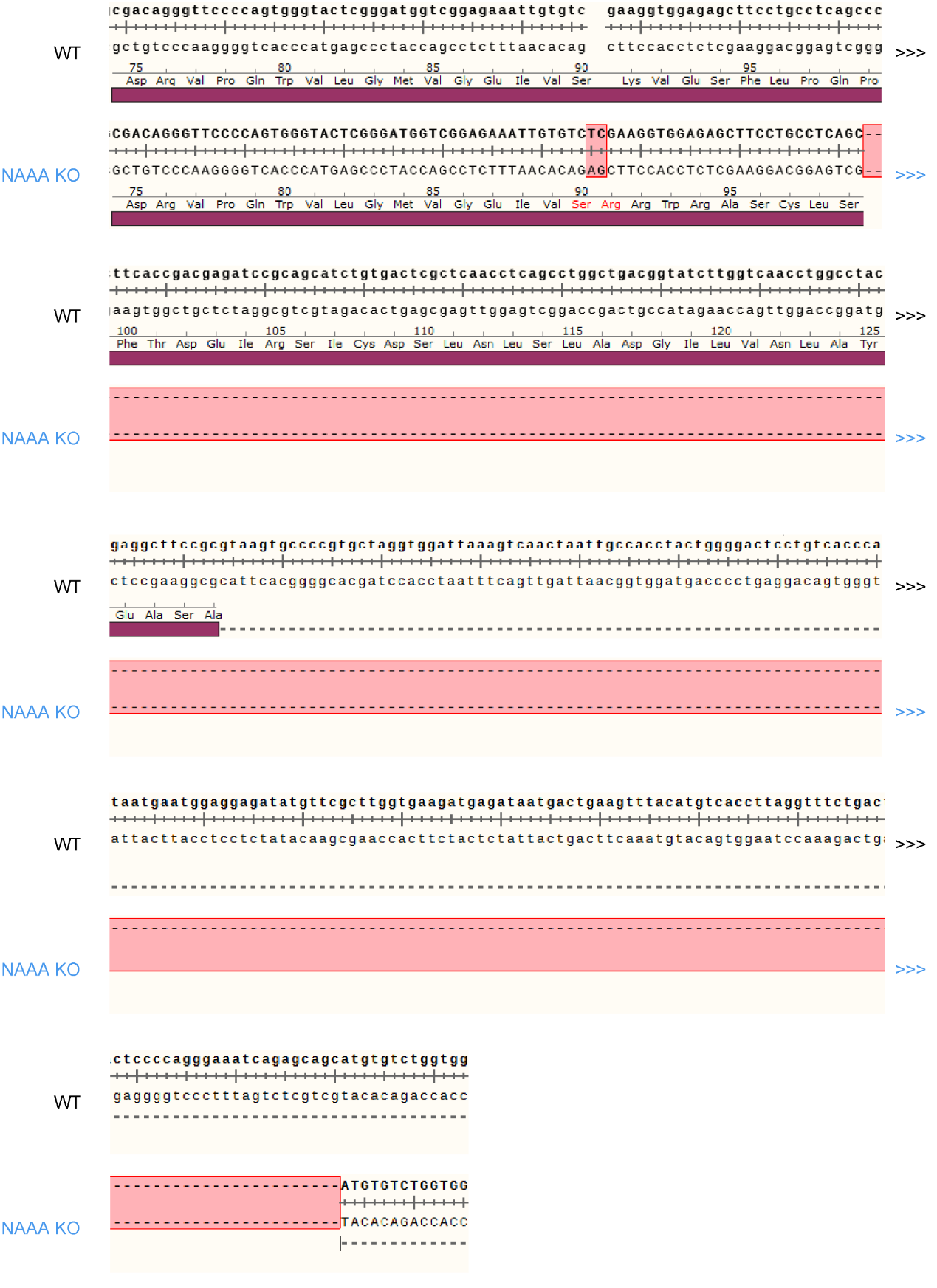


**Figure S1**. Sanger sequencing of WT and NAAA^-/-^ mice. The 2 bp insertion and 258 bp deletion are highlighted with pink boxes. The mutated exon 1 encodes amino acids 90-130 of the NAAA protein.
